# Reproductive Tract Mucus May Influence the Sex of Offspring in Cattle: Study in Cows That Have Repeatedly Calved Single-Sex Offspring

**DOI:** 10.3390/vetsci11110572

**Published:** 2024-11-16

**Authors:** Fei Huang, Peng Niu, Jieru Wang, Jiajia Suo, Lulu Zhang, Jie Wang, Di Fang, Qinghua Gao

**Affiliations:** 1 College of Life Science and Technology, Tarim University, Alar 843300, China; 107572022315@stumail.taru.edu.cn (F.H.); 107572023420@stumail.taru.edu.cn (P.N.); 107572021314@stumail.taru.edu.cn (J.W.); 107572021317@stumail.taru.edu.cn (J.S.); 2 College of Animal Science and Technology, Tarim University, Alar 843300, China; 10757223061@stumail.taru.edu.cn (L.Z.); 120240063@taru.edu.cn (J.W.); 120230093@taru.edu.cn (D.F.); 3 Key Laboratory of Tarim Animal Husbandry Science and Technology, Xinjiang Production & Construction Corps, Alar 843300, China

**Keywords:** cows, single-sex offspring, reproductive tract mucus, X/Y spermatozoa

## Abstract

This study aimed to investigate the selective effect of the reproductive tract mucus in cows that have consistently produced offspring of a single sex on X/Y spermatozoa. We collected mucus from the reproductive tract of cows that had given calvings to offspring of the same sex, or alternated between sexes, for more than five consecutive calvings. We evaluated the pH of reproductive tract mucus. Subsequently, we conducted a spermatozoa penetration assay; the proportions of X and Y spermatozoa after penetration were then identified by dual TaqMan qPCR and flow cytometry. This was followed by in vitro fertilization and embryo sex determination experiments. Immediately afterwards, computer-aided spermatozoa analysis was employed to analyze the spermatozoa that had penetrated through different types of mucus in the reproductive tract. The analysis indicated that the reproductive tract mucus of cows consistently producing male or female calves exhibited selectivity towards X/Y spermatozoa. The differences in the pH values of the reproductive tract mucus among cows continuously producing male calves, those continuously producing female calves, and those alternately giving birth to male and female calves were not significant (*p* ≥ 0.05). The outcome of dual TaqMan qPCR for cows consistently producing male calves was Y: 79.29 ± 4.28% vs. X: 21.67 ± 4.53%; for cows consistently producing female calves, the equation was Y: 25.05 ± 4.88% vs. X: 75.34 ± 5.13%. The results of flow cytometry processing revealed the following proportions: for cows consistently producing male calves: Y: 83.33 ± 5.52% vs. X: 17.23 ± 4.74%; for cows consistently producing female calves: Y: 24.81 ± 4.13% vs. X: 76.64 ± 4.21%. The outcomes of embryo sex determination for cows consistently producing male calves were as follows: male embryos vs. female embryos (79.60 ± 2.87% vs. 21.07 ± 2.51%); for cows consistently producing female calves, the outcomes for male embryos vs. female embryos were 25.58 ± 3.96% vs. 75.63 ± 3.55%. Computer-aided analysis revealed that the concentration of spermatozoa penetrating the reproductive tract mucus in cows alternating between male and female calves (9.09 ± 0.72 million/mL) was significantly higher than that in cows consistently producing male calves (6.01 ± 1.19 million/mL) and cows consistently producing female calves (5.61 ± 0.60 million/mL). There were no significant differences in spermatozoa motility, the proportion of progressive motile spermatozoa, and curvilinear, straight-line, and average path velocities. Collectively, these findings indicate that the reproductive tract mucus of cows consistently producing offspring of a single sex exhibits selectivity towards either X or Y spermatozoa. This finding is of great significance for studying the impact of maternal factors on offspring sex.

## Simple Summary

In cattle breeding, certain cows are known to exhibit a consistent pattern of producing offspring of the same sex. Our research has revealed that the reproductive tract mucus of cows that consistently deliver offspring of the same sex induces a modification in the X/Y spermatozoa proportion post-penetration. Consequently, the reproductive tract mucus of cows that consistently produce offspring of the same sex exerts a selective influence on X/Y spermatozoa.

## 1. Introduction

The sex of mammalian offspring is determined by the male gamete (X/Y spermatozoa) [1,2]. However, before conception occurs, the spermatozoa must traverse the female reproductive tract, passing through the cervix, uterus, and fallopian tubes before reaching the site of fertilization [3,4,5]. As spermatozoa travels through these regions, the mucus of the reproductive tract serves a vital function [6,7]. The mucus not only provides an appropriate milieu for the spermatozoa [8] but also supplies it with the necessary nutrition to support their journey from the vagina to the site of fertilization [9,10]. Researchers have yet to determine whether female animals have the ability to selectively favor X/Y spermatozoa during the process of fertilization.

X/Y spermatozoa are affected by multiple factors in the female reproductive tract [4,11]. For example, the pH of the diluent has varying effects on the vitality of X and Y spermatozoa [12]. When the environment is more acidic, X spermatozoa are more likely to be prevalent [13,14]. Conversely, in a more alkaline environment, Y spermatozoa tend to be more evident [12]. Furthermore, diseases of the reproductive tract that lead to an increase in cervical mucus can also affect X and Y spermatozoa [15]. An excess of cervical mucus can hinder the penetration of X spermatozoa, whereas Y spermatozoa, which typically have stronger mucus penetration capabilities, may have an advantage in such an environment [16]. The penetrating ability of spermatozoa can influence whether the spermatozoa that ultimately fertilizes is of the X or Y type. Furthermore, the presence of anti-spermatozoa antibodies in the female reproductive tract can act antagonistically against both X and Y spermatozoa, ultimately leading to infertility [17]. The membrane surfaces of X/Y spermatozoa also feature differential protein expression [18]. The presence of antibodies specific to X/Y spermatozoa in the female reproductive tract further suggests that the female can exhibit selective effects on X/Y spermatozoa.

Many phenomena of skewed sex proportions have been documented in nature, and various reasons have been proposed to account for this observation [19,20]. A known factor that causes a shift in the sex proportion of offspring is the selection of X/Y spermatozoa by the reproductive tract, although the precise mechanism involved has yet to be determined [21]. In addition, the reproductive tract can respond to spermatozoa to exhibit specific reactions to X/Y spermatozoa. For example, X or Y spermatozoa can cause differences in the transcriptome of the fallopian tube [22,23,24]; this can lead to the deposition of either X or Y spermatozoa at the bottom of the fallopian tube, thus preventing them from fertilizing the egg [25]. During the estrous period, cows produce a large amount of mucus in the reproductive tract; this mucus can exert direct effects on spermatozoa [26]. Hence, through the following specific objectives, we first assessed the pH of the reproductive tract mucus, then studied the penetration of spermatozoa through the mucosal lining of the reproductive tract in single-sex offspring cows, and detected the X/Y ratio of the penetrating spermatozoa using dual TaqMan qPCR and flow cytometry. Next, we performed in vitro fertilization and sex determination using the penetrating spermatozoa and finally analyzed the penetrating spermatozoa using a computer-assisted analysis device. The current study was designed to test the hypothesis that the mucus of the reproductive tract may exhibit selective effects on X/Y spermatozoa in cows that had previously delivered offspring of the same sex.

## 2. Materials and Methods

### 2.1. Ethics Statement

All animal experiments were conducted in accordance with the “Regulations and Guidelines for the Management of Experimental Animals” established by the Ministry of Science and Technology (Beijing, China, 2020 revision). This study was approved by the Institutional Animal Care and Use Committee of Tarim University, Xinjiang, China (protocol code DWBH20220101; approval date: 1 January 2022).

### 2.2. Animals

The experimental animals were selected from a farm in Shazhen Town, Aksu Region, Xinjiang, China (41°22′ N, 80°47′ E). The selected dairy cows for the experiment were all Holstein cows, with a body condition score (BCS) ranging from 5 to 7 [27,28] with identical feeding conditions. During the sampling period, they were fed a total mixed ration (TMR). The roughage primarily consisted of straw, fed at 10:00 and 17:00 each day. Adequate clean drinking water was provided ad libitum. Throughout the research process, the forage, feed, and feeding procedures remained relatively stable.

### 2.3. Collection of Reproductive Tract Mucus, Semen Preparation, and Collection of Oocytes

Collection of reproductive tract mucus: During the collection of reproductive tract mucus, the first step was to clean the feces from the rectum, followed by cleaning the external genital area. Then, it was necessary to gently press the reproductive tract through the rectum. The pressing sequence involved the uterine horn, uterine body, cervix, and vagina, in order. This process needed to be repeated multiple times. After several pressings, the reproductive tract mucus flowed out from the external vaginal opening. Each cow collected more than 5 mL of reproductive tract mucus, with a total of 215 mL of reproductive tract mucus collected. We collected mucus from the reproductive tract 30 min prior to artificial insemination from dairy cows that had more than five previous pregnancies resulting in offspring of the same sex (6 cows that exclusively had male calves and 6 cows that exclusively had female calves, with a control group of 6 cows that had alternating male and female calves).

Semen preparation: Throughout our experiments, we utilized conventional cryopreserved semen that was purchased at random. Commercially available frozen semen was obtained from 200 μL/tube frozen semen from straws and stored in liquid nitrogen. When thawing semen, we removed the frozen semen from liquid nitrogen and quickly placed it into a 38 °C water bath. The samples were gently shaken for 30 s; then, we tested the vitality and concentration. An effective spermatozoa count was 20 million/mL. We collected the thawed semen into a centrifuge tube for later use.

Oocyte collection: Ovaries were provided by our local slaughterhouse and transported back to the laboratory within 1 h of collection (in an incubator set at 37 °C). Then, we used a 10 mL syringe with a size 21-gauge needle to extract oocytes from follicles larger than 8 mm. In this experiment, a total of 1755 oocytes were collected.

### 2.4. Experimental Procedure

**Figure 1:**
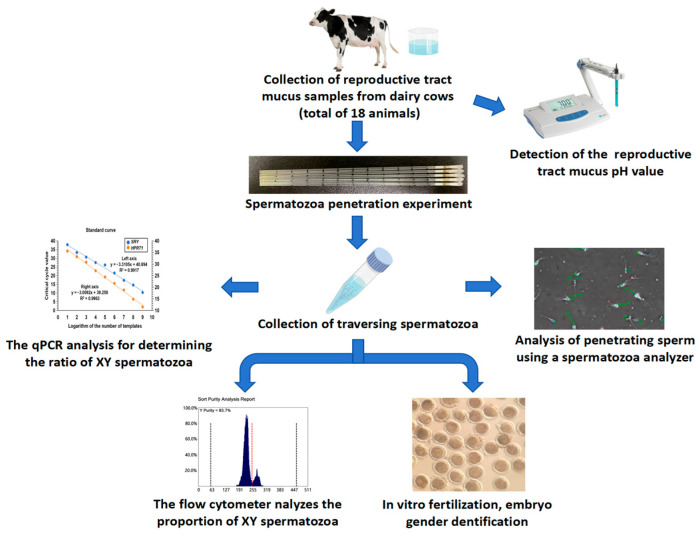
Flow chart showing the experimental procedure.

The study design is shown in Figure 1.

### 2.5. Detection of the pH Value of Reproductive Tract Mucus

The pH meter (FiveEasy Plus FE 28, Mettler ToledoInstruments Co., Ltd., Shanghai, China) was employed to measure the pH of reproductive tract mucus. Before use, the pH meter was calibrated with standard calibration solutions (pH 4.01, 7.00, and 9.21). Approximately 2 mL of reproductive tract mucus from one sample was used for pH measurement at RT. Triplicate measurements for each sample were performed.

### 2.6. Spermatozoa Penetration Experiment

**Figure 2:**
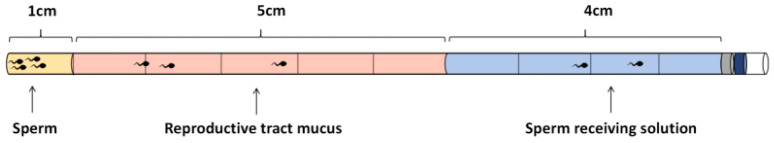
Schematic diagram showing the setup for the spermatozoa penetration experiment.

Empty semen straws were used for the spermatozoa penetration experiment; when filling with liquid, we used an empty semen straw. We connected one end to a syringe with a rubber hose and immersed the other end into the liquid that needed to be drawn in. We used a 1 mL syringe to draw the liquid into the straw. To facilitate measurement, marks were made on the semen straws at 1 cm intervals with a marker pen. First, we drew 4 cm of spermatozoa recipient medium (HTF + 5% Polyvinyl pyrrolidone) into the spermatozoa tube (Fertilization medium HTF M1130; Aibei Biotechnology Co., Ltd., Nanjing, China). Then, we introduced the reproductive tract mucus to a level that was 1 cm from the opening of the semen straws. Finally, we replenished the spermatozoa tube with seminal fluid (as shown in Figure 2). Immediately thereafter, the semen straws were placed horizontally in a 5% CO_2_ incubator for 1 h. Subsequently, at the interface between the reception fluid and the genital tract mucus, we directly cut the semen straw open. Then, we used a 1 mL syringe to slowly expel the reception fluid mixed with spermatozoa that had penetrated the reproductive tract mucus into a 1.5 mL centrifuge tube for collection.

### 2.7. Determination of the Proportion of X/Y Spermatozoa by Dual TaqMan qPCR

The determination of the X/Y proportion of spermatozoa was conducted based on the dual TaqMan qPCR method proposed by He et al. [12]. The penetrating spermatozoa were centrifuged at 500× *g* for 5 min, and the supernatant was discarded. DNA was extracted using a cell DNA extraction kit (DP304-02; TIANGEN Biotechnology Co., Ltd., Beijing, China). To calculate the X/Y proportion of the penetrating spermatozoa, we designed primers specific to the X chromosome-specific *HPRT1* gene and the Y chromosome-specific *SRY* gene, as listed in Table 1. PCR amplification was performed using genomic DNA from bovine spermatozoa as the template. The PCR products were ligated with the T-vector pMDTM 19 (6013; TaKaRa Biotechnology Co., Ltd., Beijing, China), and the resulting constructs were transformed into Escherichia coli strain DH5α competent cells using the heat shock method to prepare positive standards. By optimizing the PCR reaction system and reaction conditions, a standard curve was established to validate the specificity, sensitivity, and stability of the method. Subsequently, the proportion of X/Y spermatozoa was detected by the dual TaqMan qPCR method using a real-time fluorescence quantitative PCR instrument (LightCycler^®^96, Roche, Basel, Switzerland).

### 2.8. Assessment of the X/Y Proportion of Spermatozoa by Flow Cytometry

We collected penetrated spermatozoa into groups and centrifuged the samples (at 500 g/min) to remove the supernatant (they were divided into a continuous male calving group, consecutive female calving group, and alternate calving group), allowing the spermatozoa concentration to reach 6 × 10^8^ spermatozoa/mL. Subsequently, we diluted the penetrated spermatozoa sample to a concentration of 3 × 10^8^ spermatozoa/mL with 500 μL of diluent containing 8 μL of Hoechst 33342 dye (2 mg/mL), followed by gentle sonication for 1 s to detach the spermatozoa tails. Next, samples were incubated in a darkroom at 34 °C for 45 min and gently mixed every 10 min. We established fundamental sorting parameters, with a sheath fluid pressure of 50 psi and a laser intensity of 175 mW to ensure directional and precise spermatozoa separation accuracy at a level of >95%. We constantly monitored fluid flow, images, and other changes in the display system to promptly make adjustments and ensure the quality of sorting. A high-speed flow cytometer (SX-MOFLO, Beckman, CA, USA) was used to analyze the proportion of spermatozoa that had successfully penetrated the mucus.

### 2.9. In Vitro Fertilization and Embryo Sex Identification

A 10 mL syringe was used to aspirate follicles; the syringe contained 2 mL of oocyte extraction solution (TCM199 + 1% fetal bovine serum + 100 mg/mL sodium heparin + 100 IU/mL penicillin + 100 IU/mL streptomycin). The cumulus oocyte complexes (COCs) were then transferred to an embryo culture dish (Nunclon) containing 0.8 mL of bovine oocyte maturation medium. Next, the culture dish was placed in an incubator at 38.5 °C with 5% CO_2_ for 24 h. After 24 h of oocyte maturation, the cumulus cell layer was detached by repeated aspiration with a pipette in 0.1% (*vol*/*vol*) hyaluronidase at 38.5 °C for 1 min. Mature oocytes with the first polar body were selected and randomly divided into three groups. Spermatozoa that had penetrated the mucus of different reproductive tracts were collected in 2 mL centrifuge tubes along with the reproductive tract mucus and centrifuged at 250 g/min for 5 min. The supernatant was discarded, leaving a final volume of 20–30 μL, which was gently aspirated and mixed with a pipette. The semen that had penetrated different reproductive tracts was slowly injected into embryo culture dishes containing mature oocytes for the three groups. Subsequently, we incubated the samples in an incubator set at 38.5 °C and 5% CO_2_ for 24 h. Subsequently, the fertilized eggs were transferred to an embryo culture dish containing bovine embryo culture medium and incubated in an incubator at 38.5 °C with 5% CO_2_ until they developed into blastocysts.

Subsequently, the blastocysts were transferred into PBS for washing. Then, each individual blastocyst was placed into a 0.2 mL nuclease-free centrifuge tube, and 5 μL of genome extraction solution (50 mmol/L Tris-HCl, pH = 8.0, 0.5% Triton X-100, 1 mg/mL proteinase K) was added. Centrifuge tubes containing single blastocysts were then incubated at 37 °C for 1 h, followed by 10 min of incubation at 95 °C for lysis. We used 5 μL of post-lysis genome extraction solution as the template for the PCR. Next, we used primers for the amelogenin (*AMELY*) gene (as shown in Table 1) to conduct PCR-based gender determination on individual embryos. Subsequently, 1 μL of the first-round PCR product was used as a template for the second-round PCR. The nested PCR procedures for both rounds were identical. The PCR amplification conditions were as follows: initial denaturation at 95 °C for 3 min, followed by 35 cycles of denaturation at 95 °C for 30 s, annealing at 62 °C for 30 s, extension at 72 °C for 30 s, and a final extension at 72 °C for 5 min. Subsequently, the PCR products were subjected to 2% agarose gel electrophoresis at 100 V for 20 min, followed by visualization and photography using a gel imaging system.

### 2.10. Spermatozoa Motility Assessment

Spermatozoa motility parameters were analyzed by a computer-assisted spermatozoa analysis (CASA) system (ML-500JZ, Mairong, Guangxi, China), which included an inverted microscope (TS100-F, Nikon, Tokyo, Japan). We took 10 μL of each spermatozoa suspension that had penetrated the mucus of different reproductive tracts and slowly injected the spermatozoa into a sample chamber with a depth of 20 μm without generating bubbles. The slide was placed on a microscope stage at 37 °C. We then adjusted the brightness of the microscope illumination and displayed it on the display screen. Spermatozoa can be clearly seen, and the spermatozoa trajectory was captured at a frequency of 60 Hz (0.5 s, 30 frames). Measurements of spermatozoa concentration, the proportion of motile spermatozoa (MOT), and five CASA parameters were used for further analysis. The CASA parameters included three measures of spermatozoa velocity (curvilinear velocity [VCL], straight-line velocity [VSL], and average-path velocity [VAP]) and two measures of spermatozoa paths (linearity [LIN; VSL/VCL] and straightness [STR; VSL/VAP]). Spermatozoa were observed from at least five randomly selected fields. Three replicates per sample were examined.

### 2.11. Statistical Analysis

The statistical analysis was performed using GraphPad Prism 8.0 software. The data are presented as the mean ± standard deviation. The percentage data were transformed by arcsin square root transformation to normalize the distributions prior to statistical analysis. Differences in variables between groups were evaluated by Student’s *t*-test (two-tailed). The chi-square (and Fisher’s exact) test was used to analyze the sex rate of embryos formed by in vitro fertilization (*, *p* < 0.05; ns, *p* ≥ 0.05).

## 3. Results

### 3.1. Detection Results of pH Value of Reproductive Tract Mucus

**Figure 3:**
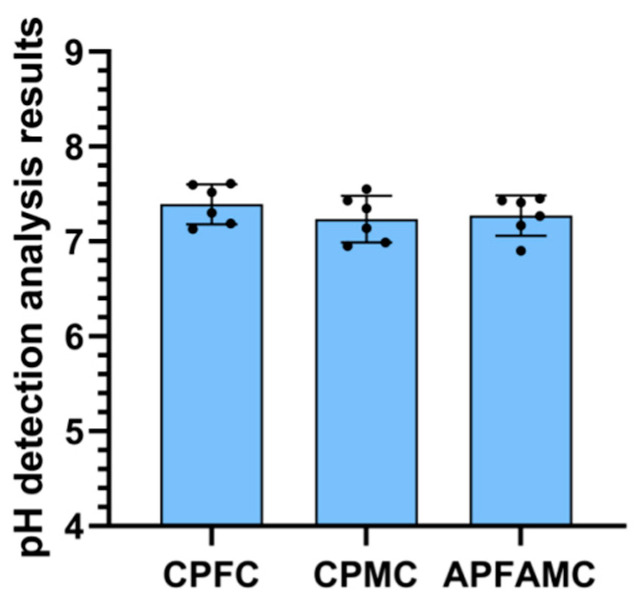
Detection results of the pH value of reproductive tract mucus. APMAFC: Alternating production of male and female calves in cows. CPMC: Cows producing male calves continuously. CPFC: Cows producing female calves continuously. The black dots represent the number of samples. ns or the absence of any notation indicates non-significant differences (*p* ≥ 0.05), while * indicates significant differences (*p* < 0.05).

The results of the pH value analysis of the reproductive tract mucus are illustrated in Figure 3. The pH value of the reproductive tract mucus in cows alternately giving birth to male and female calves was 7.27 ± 0.21; the pH value for cows continuously producing female calves was 7.39 ± 0.22; and the pH value for those continuously producing male calves was 7.23 ± 0.24. No significant differences were observed among the three groups (*p* ≥ 0.05).

### 3.2. Dual TaqMan qPCR and Determination of the Proportion of X/Y Spermatozoa

**Figure 4:**
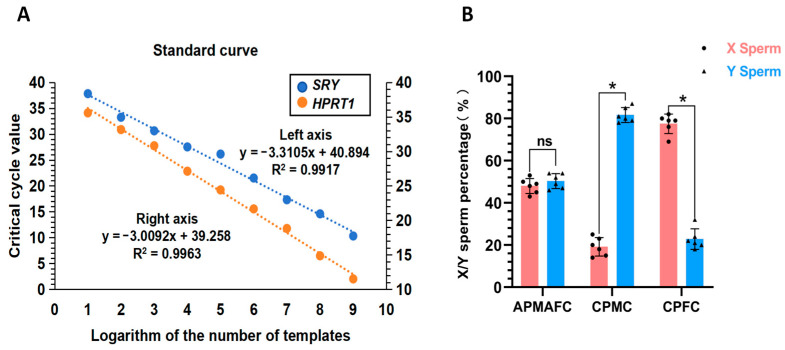
Analysis of X/Y spermatozoa proportion after mucus penetration. (**A**) represents the standard curve, while (**B**) shows the proportion of X/Y spermatozoa. ns or the absence of any notation indicates non-significant differences (*p* ≥ 0.05), while * indicates significant differences (*p* < 0.05).

Dual TaqMan qPCR was used to identify the proportion of X to Y spermatozoa after penetration. The minimum detectable levels of the *HPRT1* and *SRY* genes were 47 and 51 copies per microliter, respectively. The standard curves were as follows: y = −3.3105x + 40.894, R2 = 0.9917; and y = −3.0092x + 39.258, R2 = 0.9963 (Figure 4). Next, we calculated the X/Y spermatozoa proportion based on the Ct values from real-time fluorescence PCR amplification and the standard curves. The difference in the X/Y spermatozoa proportion in the reproductive tract mucus of dairy cows alternately producing male and female calves was not significant (X: 48.33 ± 2.08% vs. Y: 52.33 ± 2.88%; *p* ≥ 0.05). The proportion of X spermatozoa penetrating through the mucus of cows producing consecutive female offspring was significantly higher than the proportion of Y spermatozoa (X: 75.34 ± 5.13% vs. Y: 25.05 ± 4.88; *p* < 0.05). The proportion of Y spermatozoa penetrating through the mucus of cows producing consecutive male offspring was significantly higher than the proportion of X spermatozoa (Y: 79.29 ± 4.28% vs. X: 21.67 ± 4.53%; (*p* < 0.05).

### 3.3. Flow Cytometry and the Proportion of X/Y Spermatozoa

**Figure 5:**
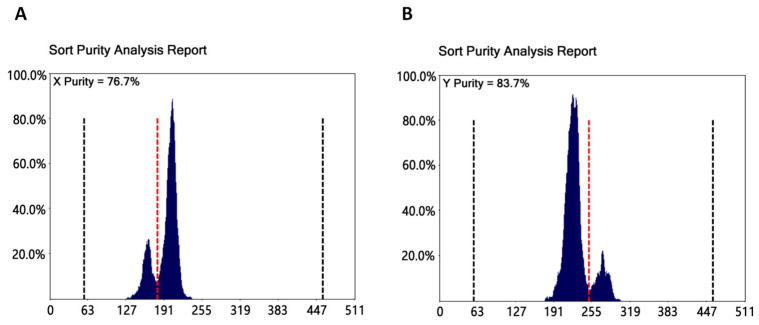
Flow cytometry results showing the X/Y proportion of spermatozoa following mucus penetration. In this figure, (**A**) represents the proportion of X spermatozoa penetrating the mucus of cows that had produced consecutive female offspring (X: 76.73%), while (**B**) represents the proportion of Y spermatozoa penetrating the mucus of cows that had produced consecutive male offspring (Y: 83.7%). The data represent the flow cytometry results from one sample, whereas the text includes data analysis from all samples, which may have some degree of error.

Flow cytometry was used to identify the proportion of X/Y spermatozoa after penetration. X and Y spermatozoa DNA was labelled with fluorescent dyes, followed by laser irradiation to identify and sort the proportions of X and Y spermatozoa (Figure 5). The proportion of X spermatozoa penetrating through the mucus of cows that produced consecutive female offspring was significantly higher than the proportion of Y spermatozoa (X: 76.64 ± 4.21% vs. Y: 24.81 ± 4.13%; *p* < 0.05). The proportion of Y spermatozoa penetrating through the mucus of cows that had produced consecutive male offspring was significantly higher than the proportion of X spermatozoa (Y: 83.33 ± 5.52% vs. X: 17.23 ± 4.74%; *p* < 0.05).

### 3.4. The Sex Proportion of Embryos Fertilized In Vitro

**Figure 6:**
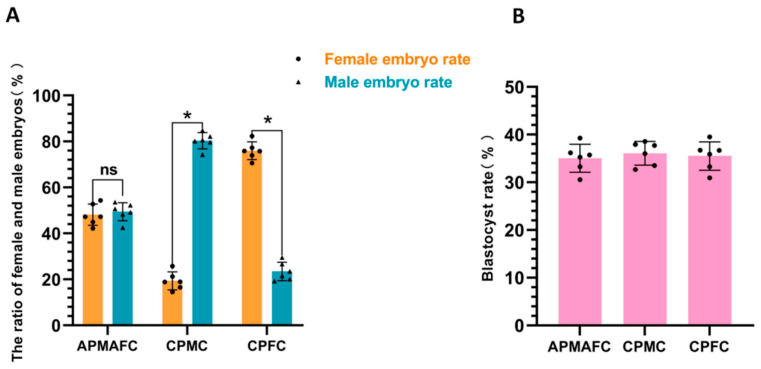
Embryo sex determination results. In this figure, (**A**) shows the results of embryo gender identification after in vitro fertilization; (**B**) shows the blastocyst rate of in vitro fertilization. ns or the absence of any notation indicates non-significant differences (*p* ≥ 0.05), while * indicates significant differences (*p* < 0.05).

Next, we used the penetrating spermatozoa for in vitro fertilization and conducted embryo sex determination once the embryos had developed to the blastocyst stage (Figure 6). A gel diagram showing sex identification in blastocysts is shown in the Supplementary Materials. Following in vitro fertilization, we found that the difference in blastocyst rates produced by spermatozoa-penetrating mucus from different reproductive tracts did not differ significantly (34.72 ± 1.57% vs. 35.86 ± 2.22% vs. 33.65 ± 2.90%; *p* ≥ 0.05) (as shown in Figure 6B). The difference in the proportion of female to male embryos produced by spermatozoa penetrating the reproductive tract mucus of dairy cows that had alternately given birth to female and male calves was not significant (female: 47.94 ± 1.87% vs. male: 52.17 ± 2.89%; *p* ≥ 0.05). Spermatozoa penetrating through the mucus of cows that had consistently given birth to male calves resulted in a significantly higher proportion of male embryos compared with female embryos (79.60 ± 2.87% vs. 21.07 ± 2.51%; *p* < 0.05). Spermatozoa penetrating through the mucus of cows that had continuously given birth to female calves resulted in a significantly higher proportion of female embryos compared with male embryos (75.63 ± 3.55% vs. 25.58 ± 3.96%; *p* < 0.05).

### 3.5. The Motility of Mucus-Penetrating Spermatozoa

**Figure 7:**
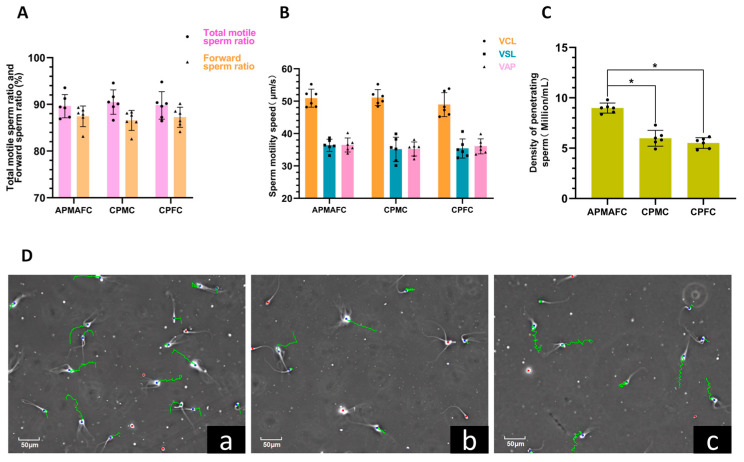
The motility of mucus-penetrating spermatozoa. In this figure, (**A**) shows the proportion of forward-moving spermatozoa and the results of spermatozoa vitality; (**B**) shows the VCL, VSL, and VAP; (**C**) shows the concentrations of mucus-penetrating spermatozoa, where the spermatozoa concentration in the APMAFC group was significantly higher than that in the CPMC and CPFC groups (*p* < 0.05), ns or the absence of any notation indicates non-significant differences (*p* ≥ 0.05), while * indicates significant differences (*p* < 0.05); In (**D**), the green line represents the trajectory of the sperm swimming, (**a**) represents the spermatozoa motility trajectory in the reproductive tract mucus of cows that produced alternate male and female offspring, (**b**) represents the spermatozoa motility trajectory in the reproductive tract mucus of dairy cows that had produced consecutive female calves, and (**c**) represents the spermatozoa motility trajectory in the reproductive tract mucus of dairy cows that had produced consecutive male calves.

The results of the analysis of mucus-penetrating spermatozoa derived from the CASA are shown in Figure 7. The concentration of spermatozoa that had penetrated the reproductive tract mucus of dairy cows that had given birth to alternating male and female calves (9.09 ± 0.72 million/mL) was significantly higher than that of spermatozoa penetrating the mucus of cows that had consistently produced male calves (6.01 ± 1.19 million/mL) and spermatozoa that had penetrated the mucus of cows that had consistently produced female calves (5.61 ± 0.60 million/mL; *p* < 0.05). There were no significant differences in any of the motion parameters of the mucus-penetrating spermatozoa, including spermatozoa vitality, progressive motility proportion, VCL, VSL, and VAP (*p* ≥ 0.05).

## 4. Discussion

Previous research has identified significant differences between X and Y spermatozoa at both the DNA level [29,30] and in terms of membrane protein composition [18]. The findings of our current research suggest and demonstrate that the reproductive tract mucus of dairy cows that consistently produce offspring of a single sex had a certain selective effect on X/Y spermatozoa. The generation of such results may be based on the differences between X and Y spermatozoa. From the moment spermatozoa enter the female reproductive tract until fertilization is complete, the environment in which X/Y spermatozoa find themselves can directly influence their ability to capacitate [31,32], their motility, and the binding of spermatozoa to the oocyte [33]. Therefore, the reproductive tract mucus of dairy cows that consistently produce offspring of a single sex may specifically affect either X or Y spermatozoa. When X/Y spermatozoa are swimming in the reproductive tract mucus, the pH of the reproductive tract mucus may affect the ratio of X/Y spermatozoa [13]. In a previous study, He et al. [14] showed that different pH levels could change the X/Y spermatozoa proportion in goats; in an acidic environment (pH = 6.2), the proportion of X spermatozoa was 67.24% ± 2.61%. Conversely, in an alkaline environment (pH = 7.4), the proportion of X spermatozoa declined to 30.45% ± 1.03%. Differences in pH can cause changes in the motility of X/Y spermatozoa [34]. However, in our study, we observed that the differences in the pH values of the reproductive tract mucus were not significant (*p* ≥ 0.05). Furthermore, the ratio of X/Y spermatozoa following penetration differed from that reported by He et al. [14]. This may be due to the special nature of the experimental animals selected in this experiment, which resulted in the difference in the ratio of X/Y spermatozoa after penetration in this experiment being greater than the results of He et al. The results of this experiment show that the Y spermatozoa penetrating the reproductive tract mucus of cows continuously producing male calves was 83.33 ± 5.52%, while the X spermatozoa penetrating the reproductive tract mucus of cows continuously producing female calves was 76.64 ± 4.21%. Multiple factors can lead to a shift in the sex proportion of offspring and quality parameters of the semen, not only including the influence of pH [35], but also ion concentration [36], different hormone concentrations [37,38], maternal obesity [39,40], and the concentration of specific binding proteins [41,42]. All of these factors can cause changes in the proportion of X/Y spermatozoa, thereby leading to a deviation in the sex proportion of the offspring. Ion concentration influences changes in the X/Y spermatozoa proportion by altering pH. The impact of hormone levels [36] and maternal obesity [39] on the offspring sex proportion was relatively minor. Antibody proteins that bind specifically can lead to a significant variation in X/Y spermatozoa proportion [43]. It was speculated from our current research results that there may be certain substances in the reproductive tract mucus that act on X or Y spermatozoa, causing the spermatozoa passing through the reproductive tract mucus to be biased toward specific types of spermatozoa.

There are different protein receptors in the tails of X/Y spermatozoa that can bind to corresponding antibodies [1,44], thereby limiting the movement of one type of spermatozoa [45], ultimately leading to changes in the sex ratio of offspring [46]. Umehara et al. [46] previously concluded that using specific receptors on the tail of X spermatozoa, the separation efficiency of bovine Y spermatozoa can reach up to 90%, and the separation efficiency of X spermatozoa can reach up to 81%. The findings of Umehara et al. [1] are similar to those observed in our current research. In this experiment, the results of dual Taq Man qPCR and flow cytometry sorting and embryo sex identification showed that the ratio of X/Y spermatozoa that penetrated different reproductive tract mucus changed significantly. Therefore, we hypothesize that there may be certain substances in the reproductive tract mucus that act on X or Y spermatozoa, leading to a change in the ratio of X/Y spermatozoa, which ultimately results in a change in the sex ratio of the offspring. The experimental animals used in this study are cows that continuously produce offspring of a single sex, which are relatively rare. This poses limitations in terms of sample collection. This study only investigated this phenomenon, and the results obtained are only preliminary results. It did not determine the mechanism that leads to the birth of consecutive male or consecutive female. In subsequent research, we will perform proteomic sequencing of the reproductive tract mucus from cows that have had consecutive male or consecutive female calvings in order to ultimately identify the proteins related to this phenomenon.

## 5. Conclusions

This study has demonstrated that the reproductive tract mucus of dairy cows producing offspring of the same sex consecutively exerts a certain selective effect on X/Y spermatozoa. In subsequent experiments, we plan to conduct physicochemical characterization and proteomic sequencing of the reproductive tract mucus from cows that have consecutively produced offspring of a single sex to more deeply investigate the reasons behind the reproductive tract mucus selective effect on X/Y spermatozoa.

## Figures and Tables

**Table 1:** Primer information for the PCR.

Primer	Sequence Form 5′ to 3′	Annealing Temperature °C	Fragment Length	Reference Sequence
*HPRT1*	Probe: HEX-CCCACTGCATCAAGCCTGGTGTTAAA-TAMRA	60	115 bp	XM_059883273.1
	F: AGCAAGCAGCTGGGATATG			
	R: TGTCTCGGTGTATGGCTAGTA			
*SRY*	Probe: HEX-TAGAAATGTCAGTTGCTGCATTCCCGA-TAMRA	60	97 bp	NM_001014385.1
	F: GTGGCCAGCTGCTTAATAGA			
	R: AGGCTCGTAGTGCAAATGAA			
*AMELY*-1	F: CATGGTGCCAGCTCAGCAG	62	X: 349 bp Y: 289 bp	NM_174240.2
	R: CCGCTTGGTCTTGTCTGTTGC			
*AMELY*-2	F: CAGCAACCAATGATGCCAGTTC	62	X: 311 bp Y: 251 bp	
	R: GTCTTGTCTGTTGCTGGCCA			

## Data Availability

The original contributions presented in the study are included in the article/Supplementary Materials; further inquiries can be directed to the corresponding authors.

## Supplementary Materials

The following supporting information can be downloaded at: https://www.mdpi.com/article/10.3390/vetsci11110572/s1. Supplementary Materials include instructions for the preparation of solutions used during the experimental process. Figures S1–S3 in the Supplementary Materials are gel electrophoresis images for the identification of embryo sex.
