# Supplementary material for: Reproductive Tract Mucus May Influence the Sex of Offspring in Cattle: Study in Cows That Have Repeatedly Calved Single-Sex Offspring
Source: Vet Sci. 2024 Nov 16;11(11):572. doi: 10.3390/vetsci11110572 (PMC11598928; doi:10.3390/vetsci11110572)
Supplement: Supplementary file 1 [file vetsci-11-00572-s001.zip › vetsci-3284170-supplementary.pdf]

veterinary sciences

MDPI

Supplementary Material

Instructions for preparing the experimental solutions used in the study.

Extract oocyte fluid

Add 0.0350 g of heparin sodium and 0.050 g of BSA to 50mL of TCM-199, mix thoroughly, and filter the culture medium with a 0.45 μm filter. Store at 4 ℃ and use within 2 weeks.

Maturation medium for bovine oocytes

Add 0.02 IU/mL FSH, 5% (vol/vol) FBS, 1 μg/mL E_2, and 0.2 mM sodium pyruvate to TCM-199. Store at 4 ℃ and use within 1 week.

Culture medium for bovine embryos

Add 2% (vol/vol) essential amino acids (BME), 1% (vol/vol) MEM, and 5% (vol/vol) fetal bovine serum (FBS) to TCM-199. Store at 4 ℃ and use within 1 week.

Electrophoresis gel images from different experimental groups.

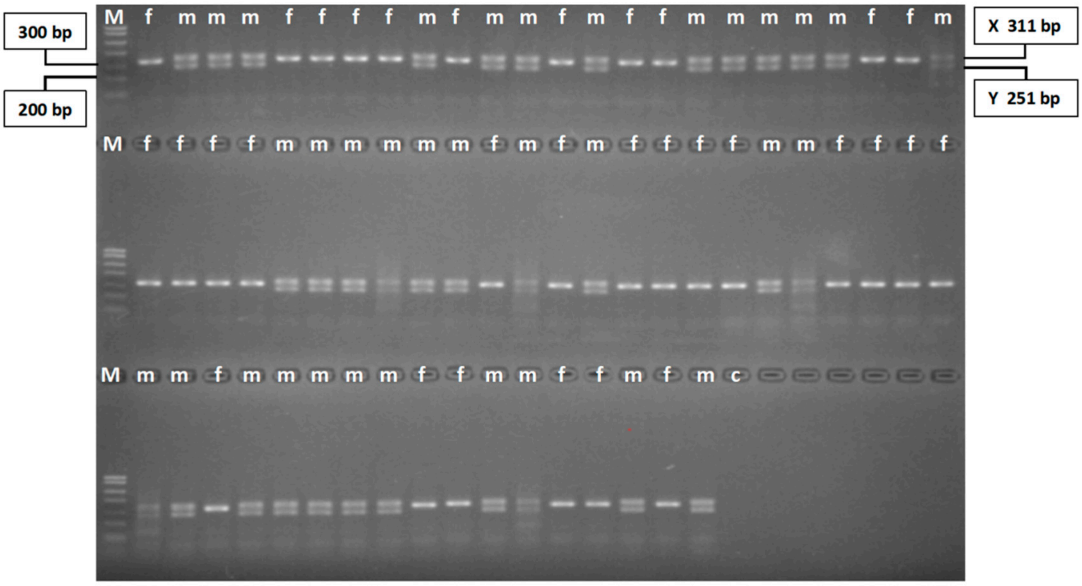

Fig.1 Results of embryo gender identification

Note: In the diagram, two bands represent male embryos, and one band represents a female embryo. The embryos resulted from spermatozoa penetration through the reproductive tract mucus of dairy cows alternating between producing male and female calves were sex-identified, with the results being 47.69% female and 52.30% male.

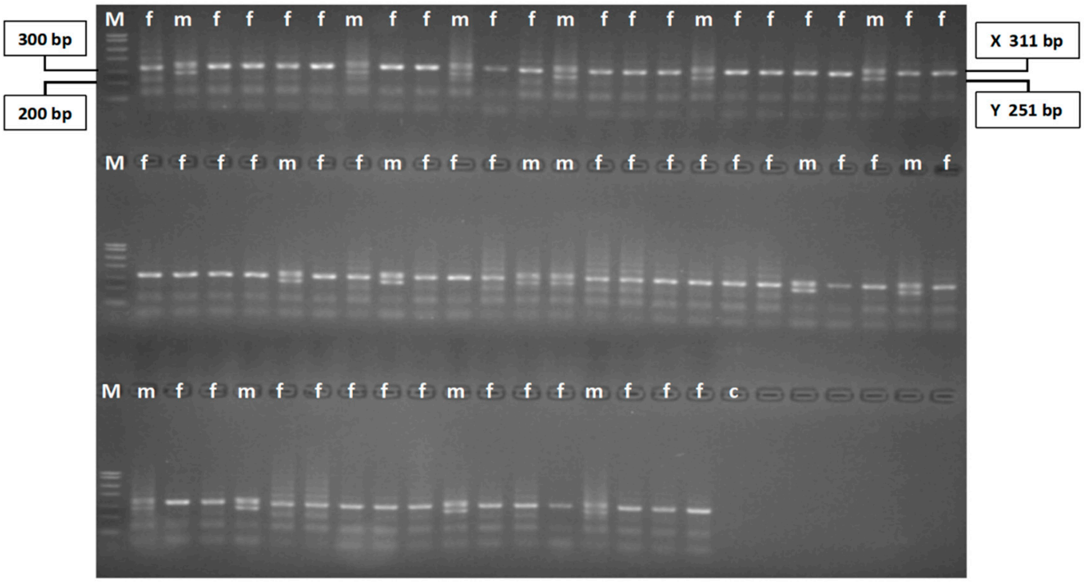

Fig.2 Results of embryo gender identification

Note: In the diagram, two bands represent male embryos, and one band represents a female embryo. The embryos produced by spermatozoa penetration through the reproductive tract mucus of dairy cows that continuously produce female calves have been sex-identified, with the results showing 75.63% female and 25.58% male.

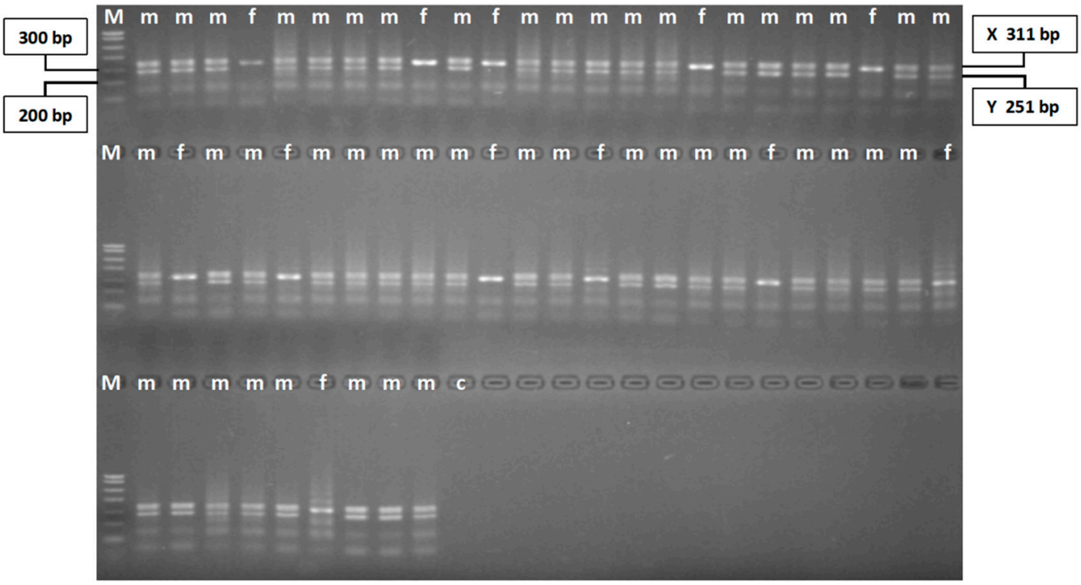

Fig.3 Results of embryo gender identification

Note: In the diagram, two bands represent male embryos, and one band represents a female embryo. The embryos produced by spermatozoa penetration through the reproductive tract mucus of dairy cows that continuously produce male calves have been sex-identified, with the results showing 20.05% female and 79.64% male.
